# Five-Year Prognosis in an Incident Cohort of People Presenting with Acute Myocardial Infarction

**DOI:** 10.1371/journal.pone.0026573

**Published:** 2011-10-20

**Authors:** Colin R. Simpson, Brian S. Buckley, David J. McLernon, Aziz Sheikh, Andrew Murphy, Philip C. Hannaford

**Affiliations:** 1 eHealth Research Group, Centre for Population Health Sciences, Teviot Place, Medical School, The University of Edinburgh, Edinburgh, United Kingdom; 2 Department of General Practice, National University of Ireland, Galway, Ireland; 3 Medical Statistics Team, Section of Population Health, University of Aberdeen, Foresterhill, Aberdeen, United Kingdom; 4 Centre of Academic Primary Care, University of Aberdeen, Foresterhill, Aberdeen, United Kingdom; Virginia Commonwealth University, United States of America

## Abstract

**Background:**

Following an AMI, it is important for patients and their physicians to appreciate the subsequent risk of death, and the potential benefits of invasive cardiac procedures and secondary preventive therapy. Studies, to-date, have focused largely on high-risk populations. We wished to determine the risk of death in a population-derived cohort of 2,887 patients after a first acute myocardial infarction (AMI).

**Methods:**

Logistic regression and survival analysis were conducted to investigate the effect of different baseline characteristics, pharmacological therapies and revascularization procedures on coronary heart disease (CHD) and all-cause mortality outcomes.

**Results:**

Within five years 44.4% of patients died (27.1% short-term [<30 days] and 23.7% longer-term [≥30 days]). Percutaneous transluminal coronary angioplasty (Adjusted Hazards Ratio (AHR) = 0.49, 95% Confidence Interval (CI) 0.26–0.93), β-blockers (AHR = 0.58, 95%CI 0.46–0.74) and statins (AHR = 0.60, 95%CI 0.47–0.77) were all associated with significant reductions in longer-term CHD-related mortality. However, not all patients received secondary preventive therapy (8.7%). Diabetes (AHR = 1.83, 95%CI 1.43–2.34), stroke (AHR = 1.73, 95%CI 1.35–2.22), heart failure (AHR = 1.69, 95%CI 1.28–2.22), smoking (AHR = 1.72, 95%CI 1.18–2.51) and obesity (>30 kg/m2; AHR = 1.39, 95%CI 1.01–1.90) increased the risk of longer-term mortality independent of other risk factors.

**Conclusions:**

It is encouraging that the coronary procedure PTCA and pharmacological secondary prevention therapies were found to be strongly associated with an important reduced risk of subsequent death, although not all patients received these interventions. Smoking, being obese and having cardiovascular related disease at baseline were also associated with an increased likelihood of longer-term mortality, independent of other baseline characteristics. Thus, the provision of smoking cessation, advice on diet (for obese patients) and optimal treatment is likely to be crucial for reducing mortality in all patients after AMI.

## Introduction

Coronary heart disease (CHD) continues to be the leading cause of mortality among adults in the industrialized world [1]. For example, it now accounts for one in three deaths in the United States of America [2]. Following a diagnosis of acute myocardial infarction (AMI), important concerns of both patients and their physicians are the subsequent risk of death and whether this can be reduced by use in real life (as opposed to clinical trials) of appropriate medical and surgical interventions.

A large multi-national study identified a number of key physical signs, biochemical measurements and co-morbidities, associated with the short-term prognosis of patients admitted to hospital for acute coronary syndrome [3]. These factors included pulse rate, systolic blood pressure, initial serum creatinine concentration, elevated cardiac markers, peripheral vascular disease and heart failure. The study, however, only followed patients for six months after their index event. Community-based studies have attempted to determine longer-term survival, sometimes up to five years, although selection procedures used when identifying the different cohorts may have introduced important biases [4]–[9]. Limitations have included: studies restricted to certain age-groups [4]–[5]; patients with specific co-morbidities such as diabetes [6]–[7]; those experiencing an emergency admission [8]; and those who are hospitalized [9]. If research is to inform everyday clinical practice, issues relating to the representativeness of samples and consequent generalizability of findings are important. A community-based perspective is especially valuable in the increasing number of countries where primary care helps provide primary prevention (to help reduce the incidence of disease and prevention of death) and much of the long-term management of chronic disease.

This paper builds on previous work, which ascertained the five-year prognosis of an incident cohort of people with angina using a novel linked primary care, secondary care and mortality database [10]. Here, we determined in routine clinical practice the five-year risk of death from any cause and CHD after a first AMI. We also ascertained whether this risk was modified by revascularization procedures and pharmacological treatments taken for secondary prevention, and (in a secondary analysis) by baseline smoking and Body Mass Index (BMI) kg/m2.

## Methods

### Sampling frame

Almost all individuals resident in Scotland in the United Kingdom are registered with a primary care practice, which provides health care services free of charge. Virtually all specialist hospital care services are also free of charge, usually obtained through referral from primary care or, in emergency situations, through patients attending an accident and emergency department. Primary care-based physicians provide, or coordinate, much of the care of patients discharged back into the community by secondary and tertiary care services.

The sampling frame for this study was all patients (n = 238,064) registered with 40 primary care practices in Scotland. Completeness of capture of contacts and accuracy of clinical event coding (using Read codes) has been found to be above 91% in these practices [11]–[12]. The electronic recording of long-term prescribing information by primary care has also been found to be accurate and complete [13]. These primary care patient data were linked in May 2007 with deprivation scores (Scottish Index of Multiple Deprivation) [14], hospital admission records for National Health Service Scottish hospitals held on the Scottish Morbidity Record (SMR) database (housed by the Information Services Division) and certified cause of death information collected by the General Register Office for Scotland (GROS). The hospital data are considered to be reliable from 1981, with completeness and accuracy rates exceeding 90% [15]. The linkage created a novel PCCIU-SMR-GROS database. We have previously shown that individuals on the linked database are broadly representative of the Scottish population, with respect to age, sex, and social deprivation [12]. Use of the de-identified linked database for this research was approved by the Caldicott Guardian for the Information Services Division.

### Incident sample

All patients who experienced an AMI apparently for the first time between 1st January 1997 and 31st December 2001 were identified. This involved identifying those whose primary care computer records contained a relevant entry of AMI (using Read codes [16]) or those with a relevant entry on the secondary care database (using International Classification of Diseases (ICD) version 10 codes I21–I22). The first such event in the timeframe was designated the index episode. The primary and secondary care data of each identified person was then checked, for as long as it existed, for any recording of an AMI prior to the date of the index episode.

### Baseline characteristics

The primary care computer records of incident cases were examined for the presence of co-morbidities and risk-factors when the index episode occurred. These co-morbidities were determined *a priori* to be associated with cardiovascular disease. The postcode of each patient was used to assign socioeconomic status on a 10-point scale, which was then converted to quintiles for analysis (1 = most affluent, to 5 = most deprived), based on the Scottish Index of Multiple Deprivation, which uses 37 indicators of poverty across seven domains (current income, employment, health, education (including skills and training), housing, geographic access and local crime statistics) [14].

As part of a subgroup analysis we also determined smoking status and BMI (kg/m2) where present in the patient record. For smoking status firstly we ascertained those that were non-smokers, then smokers and finally ex-smokers at baseline. Patients with a record of being a never smoker at any time on their primary care computer records were classified as non-smokers at baseline. If a smoking-related Read code was found prior to baseline, the patient was considered to be a smoker. If a record of an ex-smoker code was found subsequent to the latest smoking code and prior to the index date, the patient was defined as an ex-smoker. Obesity (BMI >30 kg/m2) was determined using information recorded about BMI closest to the index episode of AMI. For 242 (8.4%) patients this was recorded after the date of the index episode.

### Follow-up

Each patient was followed from the date of the index episode to death from any cause and from CHD (prior to 2000 ICD9: 410–4; thereafter ICD10: I20–25, I42, I46, I50 N.B. GROS used ICD9 for coding for slightly longer than Information Services Division) after 30 days but before five years (1,826 days, designated longer-term mortality).

We also examined the relationship between revascularization by Coronary Artery Bypass Grafting (CABG- Operation and Procedures Coding System (OPCS) version 4 code: K40–5) or Percutaneous Transluminal Coronary Angioplasty (PTCA) with or without stents (OPCS4: K49, K75, K501, K508–9), and longer-term mortality. In addition, we investigated associations between the provision of secondary prevention therapies: angiotensin converting enzyme (ACE) inhibitors, β-blockers, antiplatelets or statins, and mortality. Information about surgical procedures and medical treatments given was retrieved from the relevant SMR hospital records and primary care practice prescribing and consultation records. GROS mortality records were used to identify those who died.

### Statistical analysis

The Chi-squared test was used to test for an association between sex and other categorical baseline characteristics (age, socioeconomic status and co-morbidities). Separate statistical models were fitted for longer-term (31 days to five years) all cause mortality and CHD-related mortality.

#### Longer-term mortality

Survival analysis was conducted to investigate the effect of different baseline characteristics on time to all-cause and CHD-related mortality from 31 days to five years post-AMI. The 30-day cut-off point for mortality has been used previously [8]. We excluded patients if they died in the first 30 days of follow-up. For CHD-related mortality, patients were censored prior to five years if they died from another cause. Initially, separate Cox proportional hazards models were fitted for each baseline characteristic to determine its effect on outcome. Full models were then fitted adjusting for all potential confounders (i.e. sex, year of age, socioeconomic status and co-morbidities). Adjusted hazard ratios (AHR) and 95% CIs for each of the baseline variables are presented.

The effect of having a revascularization procedure (CABG or PTCA) and undergoing secondary prevention therapies (ACE inhibitors, β-blockers, anti-platelets and statins) during follow-up on longer-term mortality was examined by including these as time varying covariates in the survival models. Separate Cox models were fitted for each time-varying covariate and, in an extension of the adjusted mortality analysis, all six time-varying covariates were fitted together with the baseline characteristics in one model.

We examined diagnostic plots and carried out formal checks for deviation from the proportional hazards assumption using Schoenfeld residuals for each of the potential confounding variables. Kaplan-Meier curves were plotted to display the probability of survival over the study period. Analysis was conducted using SAS (v9) software package (SAS Institute, Cary, NC, USA).

#### Sub-group analysis

The above analyses were conducted on the total cohort of patients who had an AMI at baseline. As a subgroup analysis, longer-term fatality was assessed using only patients with complete data on smoking status and BMI and excluding patients with missing data (complete case analysis).

## Results

In total, 2,887 incident cases of AMI occurred during 909,097 person-years of observation, giving a crude incidence rate for AMI of 3.2 (95%CI 3.1–3.3) per 1,000 person-years. The overall mean age of patients in the study was 68.2 years (standard deviation: 13.6 years). Compared to men, women were more likely to be older and have a previous diagnosis of stroke, hypertension, atrial fibrillation or heart failure (Table 1).

**Table 1:** Key baseline variables of patients with a first diagnosis of acute myocardial infarction (n = 2,887).

		Women	Men	p-value
Baseline characteristics:		n(%)	n (%)	
**Total**		1245	1642	
**Age-band:**	<65 years	301 (24.2)	789 (48.1)	
	65–74 years	315 (25.3)	474 (28.9)	
	≥75 years	629 (50.5)	379 (23.1)	**<0.001**
**Deprivation**	1^st^	122 (9.8)	189 (11.5)	
**quintile**	2^nd^	234 (18.8)	319 (19.4)	
	3^rd^	321 (25.8)	433 (26.4)	
	4^th^	296 (23.8)	362 (22.1)	
	5^th^	272 (21.9)	339 (20.7)	0.47
**Co-morbidity**	Angina	256 (20.5)	363 (22.1)	0.34
**(yes vs no):**	Diabetes	136 (10.9)	190 (11.6)	0.63
	CKD	19 (1.5)	24 (1.5)	1.00
	Stroke	150 (12.1)	149 (9.1)	**0.01**
	PVD	95 (7.6)	125 (7.6)	1.000
	Hypertension	454 (36.5)	385 (23.5)	**<0.001**
	AF	94 (7.6)	64 (3.9)	**<0.001**
	Heart failure	161 (12.9)	129 (7.9)	**<0.001**

CKD = chronic kidney disease; PVD = peripheral vascular disease; AF = atrial fibrillation.

### Mortality

Nearly half (n = 1,282, 44.4%, 95%CI 42.6–46.2) of all the patients who had a first AMI died within five years. One fifth (n = 589, 20.4%, 95%CI 18.9–21.9) died in the community prior to hospitalization. The short-term mortality rate for patients who had a first AMI was 27.1% (95% CI 25.5–28.7; n = 783). Of the 783 patients who died within 30 days, 755 (96.4%; 95% CI 95.1–97.7) died from a CHD-related cause and were excluded from subsequent analysis.

**Figure 1:**
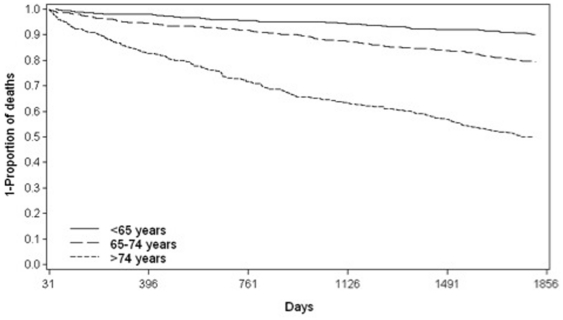
Survival function for all-cause longer term (31 days to 5 years) mortality after first acute myocardial infarction by age group (n = 2104).

Between 30 days and five years, 499 of the remaining 2104 patients died: (23.7%; 95%CI 21.9–25.5), of whom 355 (71.1%; 95%CI 67.1–75.1) died from a CHD-related cause. Amongst those who survived more than 30 days, the median time to any death was 722 (inter-quartile range (IQR) 265–1277) days, and to death from CHD 663 (IQR 225–1212) days. Older age, history of diabetes, stroke and heart failure at baseline were all associated with an increased risk of longer-term death from any cause (Table 2). Exactly the same characteristics were associated with CHD-related deaths. Figure 1 displays Kaplan-Meier curves for longer-term all-cause mortality stratified by age (<65, 65–74, 75+ years).

**Table 2:** Factors affecting longer-term (31 days to 5 years) mortality (N = 2104).

			All cause mortality			CHD mortality	
Baseline characteristic		n (%)	Unadjusted hazard ratios (95% CI)	Adjusted hazard ratios (95% CI)	n (%)	Unadjusted hazard ratios (95% CI)	Adjusted hazard ratios (95% CI)
Sex	Female	243 (28.6)	1.00	1.00	177 (20.8)	1.00	1.00
	Male	256 (20.4)	0.68 (0.57, 0.81) ‡	1.09 (0.90, 1.32)	178 (14.2)	0.65 (0.53, 0.80) ‡	1.03 (0.83, 1.29)
Age-band:	<65 years	95 (9.9)	1.00	1.00	61 (6.4)	1.00	1.00
	65–74 years	121 (20.8)	2.22 (1.70, 2.90) ‡	2.12 (1.61, 2.78) ‡	88 (15.1)	2.51 (1.81, 3.48) ‡	2.36 (1.70, 3.28) ‡
	≥75 years	283 (50.3)	6.78 (5.37, 8.56) ‡	6.25 (4.87, 8.02) ‡	206 (36.6)	7.59 (5.70, 10.11) ‡	6.83 (5.04, 9.25) ‡
Deprivation	1^st^	56 (23.1)	1.00	1.00	37 (15.2)	1.00	1.00
quintile (vs 1^st^)	2^nd^	107 (26.0)	1.18 (0.85, 1.63)	1.15 (0.83, 1.58)	69 (16.8)	1.15 (0.77, 1.71)	1.13 (0.76, 1.69)
	3^rd^	114 (20.6)	0.90 (0.65, 1.24)	0.90 (0.65, 1.24)	84 (15.2)	1.00 (0.68, 1.47)	1.01 (0.68, 1.48)
	4^th^	119 (25.5)	1.13 (0.82, 1.55)	1.17 (0.85, 1.61)	94 (20.2)	1.35 (0.92, 1.97)	1.42 (0.97, 2.09)
	5^th^	103 (24.0)	1.05 (0.76, 1.46)	1.12 (0.81, 1.56)	71 (16.5)	1.10 (0.74, 1.63)	1.16 (0.78, 1.73)
Co-morbidity	Angina	121 (27.3)	1.24 (1.01, 1.52) *	0.96 (0.78, 1.19)	95 (21.4)	1.41 (1.11, 1.78) †	1.07 (0.84, 1.37)
(yes vs no)	Diabetes	80 (35.9)	1.73 (1.36, 2.20) ‡	1.83 (1.43, 2.34) ‡	63 (28.3)	1.95 (1.49, 2.56) ‡	2.02 (1.53, 2.68) ‡
	CKD	10 (38.5)	1.98 (1.06, 3.69) *	1.76 (0.93, 3.34)	8 (30.8)	2.21 (1.10, 4.45) *	1.86 (0.91, 3.81)
	Stroke	80 (46.5)	2.50 (1.97, 3.17) ‡	1.73 (1.35, 2.22) ‡	61 (35.5)	2.69 (2.04, 3.55) ‡	1.88 (1.41, 2.50) ‡
	PVD	45 (32.6)	1.46 (1.07, 1.98) *	1.19 (0.87, 1.62)	36 (26.1)	1.66 (1.17, 2.34) †	1.31 (0.93, 1.86)
	Hypertension	156 (26.5)	1.19 (0.99, 1.44)	0.80 (0.65, 0.98) *	114 (19.4)	1.24 (0.99, 1.55)	0.78 (0.62, 0.99) *
	AF	51 (50.5)	2.76 (2.07, 3.69) ‡	1.36 (1.00, 1.86)	33 (32.7)	2.46 (1.72, 3.53) ‡	1.17 (0.80, 1.71)
	Heart failure	70 (54.7)	3.28 (2.55, 4.23) ‡	1.69 (1.28, 2.22) ‡	54 (42.2)	3.57 (2.67, 4.77) ‡	1.80 (1.31, 2.46) ‡

* p<0.05;

† p<0.01;

‡ p<0.001.

CHD = coronary heart disease; CKD = chronic kidney disease; PVD = peripheral vascular disease; AF = atrial fibrillation.

#### Revascularization

Overall, 159 (7.6%) patients who survived more than 30 days had a CABG and 222 (10.6%) had a PTCA. Ten of the 783 patients who died within 30 days of their first AMI underwent PTCA. No-one undergoing CABG died within 30 days of their first AMI. As an extension to the adjusted longer-term mortality model, cardiac procedures and secondary prevention therapy after index diagnosis (including during the first 30 days) were included as time-varying covariates. Although having a PTCA after an AMI significantly lowered the hazard of all-cause mortality, after adjusting for baseline characteristics and the other time-varying covariates, both CABG and PTCA had no significant effect (Table 3). However, a PTCA after AMI significantly lowered the hazard of CHD-related mortality, even after adjusting for baseline characteristics and the other time-varying covariates. CABG had no significant effect on CHD mortality. After PTCA, 114 (51.4%) patients had a further diagnosis of angina, 54 (24.3%) a further hospitalization for acute coronary syndrome and 13 (5.9%) for heart failure. After CABG, 60 (37.7%) patients had a further diagnosis of angina, 24 (15.1%) a further hospitalization for acute coronary syndrome and 15 (9.4%) for chronic heart failure.

**Table 3:** The effect of coronary revascularization procedures and secondary prevention therapy during follow-up (as time-varying covariates) on longer-term (31 days to 5 years) mortality.

		All-cause mortality			CHD mortality	
Time-varying covariate	n/N (%)	Unadjusted hazard ratios (95% CI)	Adjusted hazard ratios (95% CI) *	n/N (%)	Unadjusted hazard ratios (95% CI)	Adjusted hazard ratios (95% CI) *
**CABG**	10/159 (6.3)	0.88 (0.75, 1.04)	0.96 (0.81–1.14)	9/159 (5.7)	0.44 (0.22, 0.85) †	0.69 (0.35–1.35)
**PTCA**	17/222 (7.7)	0.86 (0.75, 0.99) †	0.97 (0.84–1.12)	10/222 (4.5)	0.27 (0.15, 0.51) §	0.49 (0.26–0.93) †
**ACE inhibitors**	279/1294 (21.6)	1.06 (0.97, 1.16)	1.13 (1.02–1.24) †	211/1294 (16.3)	1.55 (1.25, 1.93) §	1.74 (1.38–2.21) §
**β-blockers**	207/1364 (15.2)	0.81 (0.74, 0.88) §	0.89 (0.80–0.98) †	139/1364 (10.2)	0.41 (0.33, 0.51) §	0.58 (0.46–0.74) §
**Anti-platelets**	384/1802 (21.3)	0.98 (0.86, 1.11)	1.14 (0.99–1.32)	276/1802 (15.3)	1.03 (0.80, 1.34)	1.28 (0.96–1.71)
**Statins**	195/1456 (13.3)	0.76 (0.69, 0.83) §	0.81 (0.72–0.90) §	143/1456 (9.8)	0.41 (0.33, 0.52) §	0.60 (0.47–0.77) §

* Adjusted for baseline characteristics and all time varying covariates.

† p<0.05;

‡ p<0.01;

§ p<0.001.

ACE = angiotensin converting enzyme; AMI = acute myocardial infarction; CHD = coronary heart disease; CABG = coronary artery bypass grafting; PTCA = percutaneous transluminal coronary angioplasty.

#### Secondary preventive therapies

Of the total cohort of patients experiencing an incident AMI and surviving 30 days, 1,294 (61.5%) were issued with at least one prescription for ACE inhibitor therapy by their primary care practice, 1,364 (64.8%) for a β-blocker, 1,802 (85.6) for an antiplatelet and 1,460 (69.4%) for a statin. Some patients did not receive any secondary preventive therapy (n = 182; 8.7%) with 37.5% receiving all four (n = 788). The secondary prevention therapies of β-blockers and statins prescribed after index diagnosis were associated with a reduced risk of longer-term mortality from any cause and CHD (Table 3). Patients who were prescribed ACE inhibitor therapy during follow-up had a significantly increased risk of longer-term mortality from all-causes and CHD. Anti-platelet therapy had no significant effect on time to longer-term death. For the 1,571 patients prescribed at least one therapy (but not receiving revascularization), 597 (38.0%) patients had a further diagnosis of angina, 177 (11.3%) a further hospitalization for acute coronary syndrome and 108 (6.9%) for chronic heart failure.

#### Baseline characteristics - revascularisation and secondary preventive therapy

The baseline co-morbidities of patients receiving revascularization or secondary preventive therapies can be found in table 4. Patients receiving ACE-inhibitors had the highest rates of heart failure, atrial fibrillation, diabetes and angina at baseline. Patients receiving antiplatelets had the highest rate of stroke and were also the oldest patients. Patients receiving CABG had the highest rate of angina and peripheral vascular disease with patients prescribed β-blockers having the highest rate of hypertension.

**Table 4:** Co-morbidity of patients receiving revascularization or secondary preventive therapy (yes vs. no).

		Anginan (%)	Diabetesn (%)	CKDn (%)	Stroken (%)	PVDn (%)	Hypertensionn (%)	Atrial fibrillationn (%)	Heart failuren (%)
**CABG**	**Yes**	58 (36.5)	21 (13.2)	2 (1.3)	10 (6.3)	16 (10.1)	45 (28.3)	5 (3.1)	4 (2.5)
	**No**	386 (19.8)	202 (10.4)	24 (1.2)	162 (8.3)	122 (6.3)	543 (27.9)	96 (4.9)	124 (6.4)
**PTCA**	**Yes**	40 (16.7)	23 (9.6)	4 (1.7)	4 (1.7)	12 (5.0)	56 (23.4)	0 (0.0)	3 (1.3)
	**No**	404 (21.7)	200 (10.7)	22 (1.2)	168 (9.0)	126 (6.8)	532 (28.5)	101 (5.4)	125 (6.7)
**ACE-Inhibitors**	**Yes**	296 (22.9)	185 (14.3)	16 (1.2)	99 (7.7)	92 (7.1)	412 (31.8)	56 (4.3)	88 (6.8)
	**No**	148 (18.3)	38 (4.7)	10 (1.2)	75 (9.0)	46 (5.7)	176 (21.7)	45 (5.6)	40 (4.9)
**β-blockers**	**Yes**	304 (22.3)	142 (10.4)	8 (1.1)	80 (5.9)	77 (5.6)	395 (29.0)	45 (3.3)	52 (3.8)
	**No**	140 (18.9)	81 (10.9)	18 (1.3)	92 (12.4)	61 (8.2)	193 (26.1)	56 (7.6)	76 (10.3)
**Anti-platelets**	**Yes**	402 (22.3)	200 (11.1)	21 (1.2)	144 (8.0)	125 (6.9)	513 (28.5)	70 (3.9)	100 (5.5)
	**No**	42 (13.9)	23 (7.6)	5 (1.7)	28 (9.3)	13 (4.3)	75 (24.8)	31 (10.3)	28 (9.3)
**Statins**	**Yes**	333 (22.9)	165 (11.3)	14 (1.0)	98 (6.7)	97 (6.7)	405 (27.8)	48 (3.3)	69 (4.7)
	**No**	111 (17.1)	58 (9.0)	12 (1.9)	74 (11.4)	41 (6.3)	183 (28.2)	53 (8.2)	59 (9.1)

### Subgroup analysis

An analysis was performed on the subgroup with complete smoking and BMI data to assess whether these risk factors had an effect on mortality after first AMI (i.e. a complete case analysis). Nine hundred and two patients (31.2%) had incomplete smoking data at the time of their index event and 904 (31.3%) patients had incomplete BMI data; these subjects were excluded from the secondary analysis where smoking and BMI were included as baseline factors. Smokers had an increased risk of longer-term all-cause (AHR: 1.43 95%CI 1.05–1.95) and CHD-related mortality (AHR: 1.69 95%CI 1.16–2.46), compared with non-smokers. Ex-smokers were no more likely to have longer-term mortality after first AMI than non-smokers (AHR: 0.96 95%CI 0.69–1.35). Obese patients had a significantly higher hazard of all-cause mortality compared to non-obese patients (AHR: 1.39 95%CI 1.01–1.90).

## Discussion

### Summary of main findings

Nearly half of all the patients in this large community-based cohort who had a first AMI died within five years. More than a quarter of patients died within 30 days of having their first AMI, mostly (96.6%) from CHD causes. Patients who survived to at least 30 days after their first AMI and who received PTCA or some (but not all) secondary preventive treatments after the diagnosis of AMI, had important reductions in their risk of subsequent mortality. The risk of longer-term mortality amongst smokers and the obese was substantially increased, independent of other risk factors such as socioeconomic status and age.

### Strengths and limitations of this work

A major strength of our study was the use of a large incident cohort of patients identified using both primary and secondary care records. Other strengths were the length of follow-up, the availability of data on a number of characteristics from a representative sample of community dwelling individuals prior to their first AMI and the novel database linkage that allowed the use of data recorded in secondary as well as primary care, and cause of death data from death certificates. By combining primary and secondary care data, we were able to identify and exclude patients who were likely to have had a previous history of AMI before the index episode. The use of outcome data from secondary care and GROS also allowed us to follow up individuals even if they left their primary care practice after the index episode, since all hospitalizations (in Scotland) and mortality data were available to us.

Although we were able to include a number of co-morbidities and risk factors in our models, some clinical, electrocardiographic, and enzyme or biomarker characteristics used to help guide triage and immediate management decisions were not available. Other limitations include absence of information about the use of thrombolytics and other effective cardiac medications used in hospital. We did not have any details of the criteria used when recording an episode of AMI, or of any investigations used to make the diagnosis. It is possible that different criteria were used for different groups within the population (especially for events leading to death before hospitalization), although the strong relationship between baseline characteristics and fatal outcome provides some face validity for the index diagnosis. As some co-morbidities were relatively uncommon, such as chronic kidney disease, we sometimes lacked power to reliably detect statistical significant relationships that may exist.

### Considering the findings from this work in relation to other published literature

In terms of its baseline characteristics, our community-based cohort differed in several ways from previous cohorts used for prognostic studies. Although our cohort had a similar age and sex profile as a cohort of patients admitted to acute care general hospitals with AMI in Worcester, USA between 1995 and 2001 (68 years vs. 69 years; male patients 58% vs. 58% respectively) [9], the medical history profile of the two cohorts was quite different. For example, 31% of individuals in our study had hypertension, 11% diabetes, and 11% heart failure, compared with 69%, 32% and 22% respectively in the Worcester cohort. Rates of CABG and PTCA found in our cohort were lower to rates reported amongst survivors in the Worcester cohort (CABG: 7.6% vs 11.2%; PTCA: 10.6% vs. 35.5%) [17]. This may reflect lower rates of revascularization procedures performed in Scotland at the time, which may have contributed to higher subsequent mortality.

Another large global cohort of patients with acute coronary syndrome identified in cardiac clinics included patients with a prior history of AMI (the GRACE trial) and has subsequently been used to calculate a hospital mortality risk score [18]. The GRACE cohort had a similar age profile (65.0 vs. 68.2 years), but a higher proportion of males (66.0% vs. 56.8%) than our cohort. Our short-term fatality of 27.1% was higher than the 19.9% found using a Scottish national hospital based cohort [8], but similar to fatality rates found in a cohort of Swedish first AMI patients, which also included pre-hospital deaths (23.5% to 23.8%) [19]. Our longer-term mortality rates were similar to those found from Scottish national datasets (23.7% vs. 23.3%) [8].

Our population presenting with a first AMI also differed from the general Scottish population [20]. For instance the rate of diabetes in our population was higher (women: 10.9% vs. 3.7%; men: 11.6% vs. 3.8%) [21]. Rates of hypertension were higher for women (36.5% vs. 33.0%) but lower for men (23.5% vs. 31.7%). Smoking rates in our cohort (women: 38.5% and men: 49.5%) were much higher than the general population (2003 - women: 28% and men: 29%). In contrast, rates of obesity (women: 17.1% and men: 15.4%) were lower than the general population (2003 - women: 26% and men: 22.4%) although this is possibly due to the older age of our population.

Differences between these other cohorts and ours – which included as far as possible every incident AMI over five years amongst nearly a quarter of a million people living in Scotland – highlight the need for caution when considering the application in community settings of risk scores or guidelines developed in other settings [18], [22]. However, it is still probable that the impact of risk factors and secondary prevention on subsequent mortality in our cohort would apply equally, if not more so, in populations without AMI and those outside of Scotland [10].

Our finding of a significant association with smoking at baseline and an increased risk of longer-term all-cause and CHD death does not support the previously observed ‘paradox of smokers’ surviving longer post-AMI than non-smokers [23]. A number of baseline co-morbidities were associated with subsequent outcome. The association in our study between diabetes at baseline and subsequent long-term fatality is in line with previous findings from a recent primary care cohort of people with CHD [24] and our work using these linked data [10]. Contrary to previous findings, we observed an increased risk of longer-term mortality among those receiving ACE inhibitors (AHR 1.13, 95%CI 1.02–1.24 vs. relative risk 0.87, 0.81–0.94 [25]) and no significant effects among those receiving anti-platelets (AHR 1.14, 0.99–1.32 vs. risk ratio 0.79, 0.74–0.84 [26]). It is plausible that these results could be due to confounding by indication. With a relatively poor prognosis [27] and an increased likelihood of subsequent all-cause and CHD-related mortality being found for this cohort, heart failure was found in proportionally more patients receiving ACE-inhibitors than those that didn't receive the therapy. For other therapies, proportionally more patients did not have a history of heart failure and a better prognosis. Although we have attempted to account for markers of disease severity such as patient's baseline characteristics, further work is required to identify markers of disease severity from routinely collected datasets, which can be used to take into account the propensity to be prescribed drugs or given an intervention.

### Conclusions

Our study has provided an innovative opportunity to consider the longer-term mortality risk observed in routine clinical practice amongst a community-based incident cohort of people who experienced their first AMI in Scotland. It is encouraging that the coronary procedure PTCA, and pharmacological secondary prevention therapies were found to be strongly associated with an important reduced risk of subsequent death, although an opportunity exists for optimization of medical therapy as not all patients received these interventions. In addition, smoking and being obese at baseline were associated with an increased likelihood of mortality, independent of other baseline characteristics. Thus, it is likely (with the proviso that these data can only show association rather than causation of risk factors and outcome) that the provision of smoking cessation, advice on diet (for obese patients) and optimal medical therapy are crucial for reducing mortality in all patients after AMI. Our study also demonstrates that linked clinical datasets such as this one provides an important opportunity to study prognosis quickly and cost-effectively, and assess (all be it without being able to “trump” trial findings) the likely generalizability of trial findings when implemented into routine care.
